# Temperature Sequential Data Fusion Algorithm Based on Cluster Hierarchical Sensor Networks

**DOI:** 10.3390/s20164533

**Published:** 2020-08-13

**Authors:** Tianwei Yang, Xinyuan Nan, Weixu Jin

**Affiliations:** School of Electrical Engineering, Xinjiang University, Urumqi 830047, China; bebetterytw202416@163.com (T.Y.); j1429175857@163.com (W.J.)

**Keywords:** sensor information fusion, clustered sensor networks, fading memory matrix, sequential analysis, inverse covariance intersection

## Abstract

The process of extracting gold by biological oxidation involves oxidizing the refractory high-sulfur and high-arsenic ore with the help of bacteria to decompose the wrapping material of gold to extract the gold. Therefore, maximizing the activity of bacteria will directly affect the efficiency of gold extraction, for which it is particularly important to maintain the pulp temperature in the oxidation tank at the optimal bacteria breeding temperature. However, gold mines are generally located in mountainous areas, and the large temperature difference between day and night in winter, coupled with the influence of wind and snow, creates variations in the temperature in the oxidation tank. The traditional temperature measurement method cannot fully reflect the temperature change of the oxidation tank. As a multi-field application method, sensor information fusion can effectively address the problem of pulp temperature measurement. First, we analyzed the heat transfer principle inside the oxidation tank, and designed the cluster hierarchical sensor network according to the spatial position of each oxidation tank and the environmental interference factors. The network structure is divided into three layers; the bottom of the sensor to collect pulp temperature data shows a spiral distribution in the inner wall of the oxidation tank. Each cluster head node sensor is used as an intermediate layer to complete local measurement fusion estimation. Finally, the fusion center is taken as the upper layer to realize the global state fusion estimation. Secondly, in the data processing of the bottom temperature sensor, the traditional unscented Kalman filter (UKF) algorithm is improved and the fading memory matrix is added to improve the identification of nonlinear modeling errors. The sequential observation fusion estimator (SOFE) algorithm is embedded in the measurement update to improve the performance of local measurement fusion. Finally, in the global state fusion estimation, the sequential analysis is combined with the inverse covariance intersection, and the sequential analysis and inverse covariance intersection-global state fusion estimation (SICI-GSFE) algorithm is proposed. Through calculation and simulation, the results show that the external interference can be reduced by combining all the temperature state estimations, and the accuracy of the best global temperature state estimation is improved.

## 1. Introduction

**Figure 1:**
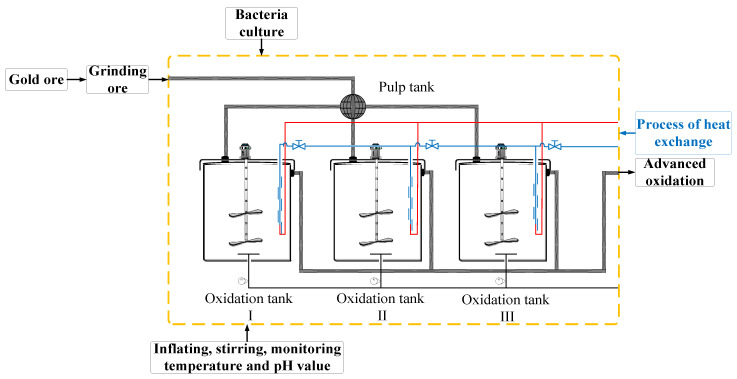
Biological oxidation pretreatment process.

The versatility of low-cost wireless sensor networks and the diversity of multi-sensor fusion applications have aroused increasing research interest in the past decade, including for air target tracking and spacecraft navigation in military fields, and environment monitoring and video image processing in the civil field. As a new technology in the process of extracting gold from refractory ores [1], biological oxidation pretreatment can expose gold through the pretreatment of gold inclusion by bacterial oxidation. To improve the gold extraction rate, it is necessary to ensure the bacterial activity, and temperature is an important factor affecting bacterial activity and reproduction [2]. Therefore, research on using wireless sensor networks to monitor the temperature parameters in the biological oxidation gold extraction process to improve the accuracy is needed. The biological oxidation pretreatment process is shown in Figure 1: The interior of the dotted line is primary oxidation and the structure adopts No. I, II, and III oxidation tanks in parallel. The diluted pulp is introduced by the self-adjusting propeller tank, and nutrients and sulfuric acid are added at the same time. When each index in the oxidation tank meets the bacterial activity requirements, cultured bacteria are added for oxidation pretreatment.

### 1.1. Related Works

As the multi-sensor fusion method that improves sensor accuracy and provides more accurate decision-making is widely used in various research fields, Varshney [3] designed and analyzed a distributed detection system. The decision rules of different detectors under various detection criteria were determined. Rossi [4] reported that wireless sensor networks, as a typical solution for data acquisition, data processing, and decision-making, have been widely used in different research fields. Hall [5] introduced the multi-sensor data fusion technology used in the Department of Defense (DoD) field of the Ministry of Defense and non-Ministry of Defense, analyzed the process model, and commented on the development prospects of data fusion technology, providing a basis for future research and applications of data fusion technology. From a macro point of view, the research results of combining perceptual data through algorithms are better than using sensors alone. For example, Garcia and Martín [6], to surmount the limitations of a single sensor, designed a multi-sensor fusion method based on computer vision, a laser scanner, and a global positioning system for intelligent vehicles to make the road environment safer. Pham [7] used a multi-sensor data fusion algorithm to monitor the accidental falls of firefighters during a fire and saves their lives by calling the police in time. In a previous study [8], a home-based wireless electrocardiogram (ECG) monitoring system using Zigbee technology was considered. Real-time monitoring systems record, measure, and monitor the heart electrical activity while maintaining consumer comfort. 

Data fusion technology has also been combined with many aspects of research, such as filtering technology, neural networks, and clustering analysis. For example, Safaria and Shabani [9] analyzed and studied the problem of multi-rate and multi-sensor data fusion based on a linear system and proposed a method using a neural network to estimate the state vector of the filter output of each sensor system. Cappello [10] proposed a multi-sensor data fusion technology based on a particle filter (PF) and compared it to other technologies in a small long-range aircraft system; the position, speed, and attitude measurement performance were better in the proposed system. Turan [11], to overcome the attitude positioning problem of an endoscopic capsule robot, proposed a multi-sensor data fusion method based on a PF and a recurrent neural network. Zhang [12] designed a multi-source data fusion model based on a fuzzy neural network to monitor the real-time workload of pilots and facilitate the timely warning of workload imbalance in the cockpit. The performance of this method was found to be better than those of other data fusion methods. 

Regarding decision fusion involved in distributed detection in sensor networks, Zhang [13] studied the distributed detection problem with dependent sensor decisions. To overcome the limitation of the existing standard multivariate copulas, an optimal fusion rule using a regular vine copula was proposed under the Neyman–Pearson framework. Ciuonzo [14] studied the problem of distributed detection of non-cooperative targets using wireless sensor networks. Developed fusion rules based on generalizations of the well-known locally optimum detection (LOD) framework: Bayesian-LOD and generalized-LOD.

The problems of information transmission and energy consumption in wireless sensor networks are also research hotspots for multi-sensor data fusion at present. For example, Li [15], to solve the problem of distributed detection of sparse stochastic signals with one-bit data, proposed the improved one-bit locally most powerful test (Im-1-bit LMPT) detector to detect sparse signals, which compensates for the performance loss caused by one-bit quantization. Cheng [16], considered a wireless sensor network (WSN) model that includes intelligent and non-intelligent sensors, and proposed the Rao test for decentralized detection of an unknown deterministic signal in WSNs sign zero-mean, unimodal, and symmetric noise. Rossi [17] exploited the time correlation of the unknown binary source and proposed a new decision fusion (DF) method. Aiming at the WSN with randomly deployed sensors, Ruixin [18] studied the performance of the counting rules for hypothesis testing using the total number of tests reported by local sensors in the fusion center. Ciuonzo [19] analyzed the channel-aware binary-decision fusion over a shared Rayleigh flat-fading channel with multiple antennas at the Decision Fusion Center (DFC) and solved the issues related to fixed point implementations and reduced WSN energy budget.

To overcome the limitations of a single sensor in regional environmental monitoring, multi-sensor data fusion technology has been widely used in many environmental target monitoring processes [20,21,22,23].

The goal of this study was to solve the problems related to the temperature inspection and estimation of the sensor. In some recent studies related to temperature detection, aiming at the problem of regional ambient temperature detection, Lee [24] analyzed and compared the accuracy of error- and entropy-based sensor layouts in determining the best sensor location for monitoring and controlling the environment in the greenhouse. Poma [25] developed an autonomous system for remote monitoring of sea water temperature and pH to evaluate the current situation of the marine environment and predict future changes. Runkle [26] studied a method based on wearable sensors to detect the ambient temperature of outdoor workers to estimate the correlation between multiple heat stress events and temperature changes experienced by individuals. In other studies [20,27,28], Yang proposed an adaptive weighted fusion algorithm to search the minimum standard deviation, which enabled the measurement of molten pool temperature using a multi-sensor measurement system. Aiello proposed an intelligent power management decision support system based on wireless sensor networks. A sensor data fusion algorithm was deployed in the system to detect the change in greenhouse ambient temperature and optimize global decision-making, which helps reduce the use of pesticides and fertilizers in protected crops. These research results were a primary consideration for our design of a fusion algorithm for temperature data collected by sensors.

The objectives of the above research primarily focused on specific equipment temperature detection and regional ideal ambient temperature detection, such as in high-temperature molten pools [27] and permanent crop greenhouses [20]. A specific algorithm was applied to measure the temperature data inside the greenhouse at nine sensor positions to determine the optimal sensor location [24]. 

### 1.2. Problem Statements

**Figure 2:**
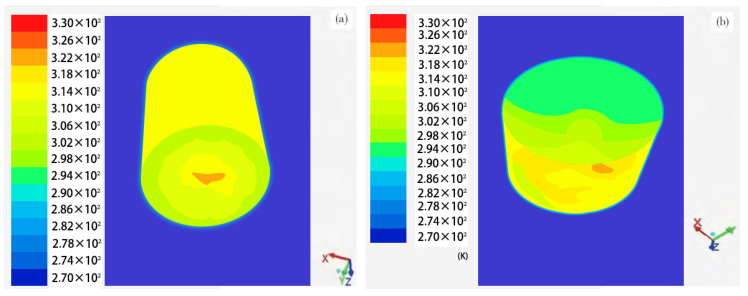
Temperature field in (**a**) normal and (**b**) extreme climate.

Our research is related to the biological oxidation pretreatment process and equipment. The temperature measurement target environment is different from the previous research, which mainly focused on the following three points: First, due to the bio-oxidation extraction of gold from refractory minerals (including pyrite, pyrrhotite, and arsenic-bearing minerals), based on the characteristics of minerals, most of the industrial sites are high-altitude mountainous areas, which are greatly affected by the environment. Second, biological oxidation pretreatment process equipment (oxidation tanks) are large and usually placed outdoors. Third, Lee [24] analyzed the problem of redundant information in the sensor measurement data. The smaller the amount of redundant data, the larger the amount of effective information for temperature measurement, which is directly related to the distribution of the sensor position. For the purpose of cost savings, the commonly used measurement schemes in the actual biological oxidation gold extraction industry are the installation of a single sensor measurement or using manual hand-held sensors for contact measurement. When the oxidation tank is set-up in an outdoor high-altitude area, it is influenced by numerous interfering factors. Therefore, temperature detection is not accurate. In the aforementioned study, our laboratory analyzed the distribution of the temperature field in the oxidation tank in detail. Ning [29] reported the temperature distribution inside the bio-oxidation tank under diverse climatic conditions (Figure 2). Due to the extreme weather at high altitudes, the temperature distribution inside the oxidation tank is uneven, which affects the activity of oxidizing bacteria, thereby reducing metallurgical output.

To ensure the sensor can accurately monitor the pulp temperature in the pretreatment oxidation tank and improve the accuracy of global state estimation of the temperature of the pretreatment oxidation tank, a hierarchical data fusion algorithm for clustered sensor networks that can adapt to the influence of a high-altitude environment is proposed. 

The main contributions of this study are as follows:

(1) The internal structure and heat transfer principle of the oxidation tank in the biological oxidation pretreatment process are studied, and the previously established state model is analyzed to provide the basis for the proposed fusion algorithm. 

(2) Based on the unbalanced distribution of the internal temperature field in the oxidation tank under the influence of an extreme environment, a hierarchical data fusion structure of a clustered sensor network is designed that agrees with the global monitoring of temperature change in a multi-stage oxidation tank. The division of labor at each level of the sensor is clear, including temperature acquisition, original data processing, and global data fusion. The sensor level from low to high depends on the installation position inside the oxidation tank. The temperature data collected by the bottom-level sensors play an important role in the global temperature state estimation of the fusion center. 

(3) Considering the state estimation of a multi-stage oxidation tank, the traditional unscented Kalman filter (UKF) algorithm is improved in terms of bottom data acquisition and the local estimation of a single-stage oxidation tank. First, the idea of sequential measurement fusion (SMF) estimation is introduced, and then combined with the measurement update link after the unscented transform (UT). The locally fused measurement estimation is obtained.

(4) The temperature state data collected by the bottom sensor in the multi-stage oxidation tank are sent to the global fusion center. Based on the matrix-weighted global state fusion estimation (GSFE) algorithm, the idea of the intersection of sequential measurement and inverse covariance is introduced to reduce the computational cost and improve the estimation performance. 

(5) Compared to the traditional oxidation tank pulp temperature measurement method, the proposed multi-stage oxidation tank clustering sensor fusion method can estimate the global state of in-tank temperature measurement in a high-altitude environment. 

The rest of this article is organized as follows: Section 2 introduces the heat transfer mechanism and fusion framework of the oxidation tank, and Section 3 describes the proposed algorithm in detail, including the improvement in the traditional unscented Kalman filter algorithm and the design of the hierarchical fusion algorithm. In Section 4, the proposed algorithm is tested and simulated to verify its performance. Section 5 summarizes the study and recommends future work and the corresponding solutions.

For a random variable $x\in R^{n}$, $A^{T}$ represents the transposition of A, |A| is the determinant of A, diag(…) denotes the diagonal matrix whose diagonal elements are the entries in (.), and E{.} or Cov[.] denotes the mathematical covariance. Notation used in this study are shown in Table 1.

## 2. Heat Transfer Mechanism and Fusion Frame

### 2.1. Heat Exchange Analysis of Oxidation Tank

**Figure 3:**
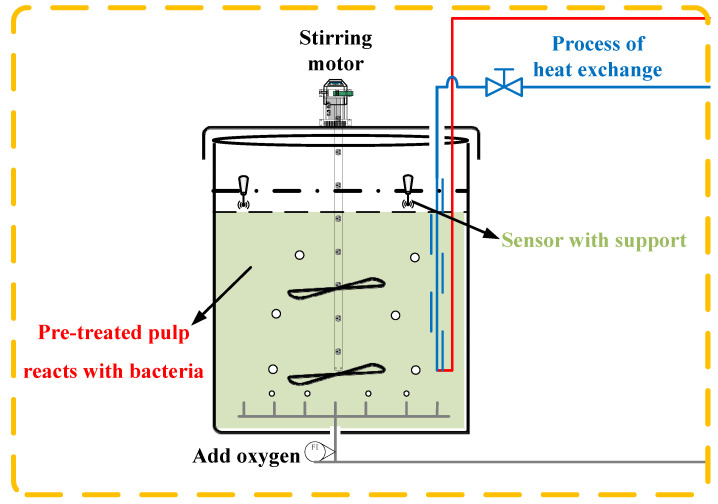
The basic structure of the oxidation tank.

In the process of biological oxidation pretreatment, the pulp temperature is controlled by adjusting the flow rate of cold/hot water in the serpentine heat exchanger in the tank to ensure the best bacterial activity. Figure 3 shows the basic structure of the independent oxidation tank. 

When analyzing the heat exchange process between the heat exchanger and the pulp in the oxidation tank, the related heat transfer mechanism accords with the Fourier convection heat transfer law [30]:

(1) $$-\beta \frac{\partial \Psi_{h}}{\partial s}=h(\Psi_{h}-\Psi_{o})$$

where β is the heat conduction coefficient, $\Psi_{h}$ is the temperature of the serpentine heat exchanger, $\Psi_{o}$ is the temperature of the pulp, h is the heat transfer coefficient, and s is the coordinate of a certain point at the junction of the heat exchanger and the pulp.

The heat in the serpentine heat exchanger is transferred into the pulp, and its transfer process follows the law of conservation of energy, which is given by the following heat exchange differential equation:

(2) $$-\rho \mathbb{C}\left(\frac{\partial \Psi }{\partial t}+d_{x}\cdot \frac{\partial \Psi }{\partial x}+d_{y}\cdot \frac{\partial \Psi }{\partial y}+d_{z}\cdot \frac{\partial \Psi }{\partial z}\right)=\left(\beta_{x}\frac{\partial^{2}\Psi }{\partial x^{2}}+\beta_{y}\frac{\partial^{2}\Psi }{\partial y^{2}}+\beta_{z}\frac{\partial^{2}\Psi }{\partial z^{2}}\right)+\gamma +\omega$$

where ρ is the density; ℂ is the specific heat capacity; t∈[0,T] is the time; $d_{x}$, $d_{y}$, and $d_{z}$ represent the displacement components along the x, y, and z directions, respectively; $\beta_{x}$, $\beta_{y}$, and $\beta_{z}$ represent the heat conduction coefficients along the x, y, and z directions, respectively; Ψ is the temperature; γ is the heat source (heat exchanger); and ω is the temperature white noise, ‖ω‖≪‖γ‖.

As the outdoor environment is at high altitude, affected by strong wind, severe cold, and other factors, heat loss will occur on the inner wall of the oxidation tank, which can be expressed by

(3) $$Q_{l}=h_{l}\left(\Psi_{o}-\Psi_{l}\right)$$

where $Q_{l}$ is the heat loss per unit area per unit time, $\Psi_{l}$ is the inner wall temperature of the oxidation tank, and $h_{l}$ is the heat transfer coefficient between the pulp and the tank wall. 

When the temperature reaches the process setting, the temperature stability is maintained when the heat gain and loss of the oxidation tank per unit time are balanced.

The internal space of the oxidation tank is discretized, and the pulp temperature change with time is mainly related to the pulp temperature with time, the heat exchanger, and the extreme external environmental changes. Based on this, the discrete equation of the state of the pulp heat exchange in the oxidation tank at time *k* is expressed as

(4) $$X_{k}=\left[\begin{array}{c} \psi_{k}\\ \gamma_{k} \end{array}\right]=\left[\begin{array}{cc} A_{k-1} & \Delta t\cdot I\\ 0 & A_{k-1}^{’} \end{array}\right]\left[\begin{array}{c} \psi_{k-1}\\ \gamma_{k-1} \end{array}\right]+\left[\begin{array}{c} \omega_{k-1}\\ \eta_{k-1} \end{array}\right],k=0,1,\ldots \ldots$$

where $\psi_{k}$, $\gamma_{k}$, $\omega_{k}$, and $\eta_{k-1}\in R^{n}$, Δt is the time step, and $\eta_{k}$ is the white noise at the heat source. It is assumed that the heat source is only affected by white noise; thus, $A_{k-1}^{’}=I$. At each time step, a sensor is used to measure the pulp temperature inside the oxidation tank. The equation of time measurement can be expressed as

(5) $$Z_{\left(i,k\right)}=C_{\left(i,k\right)}^{T}\psi_{k}+v_{\left(i,k\right)},i=1,2,\ldots ,N$$

where $C_{\left(i,k\right)}\in R^{n\times m}$ and $v_{\left(i,k\right)}$ is the white noise of the measurement process.

### 2.2. Design of Fusion Framework

**Figure 4:**
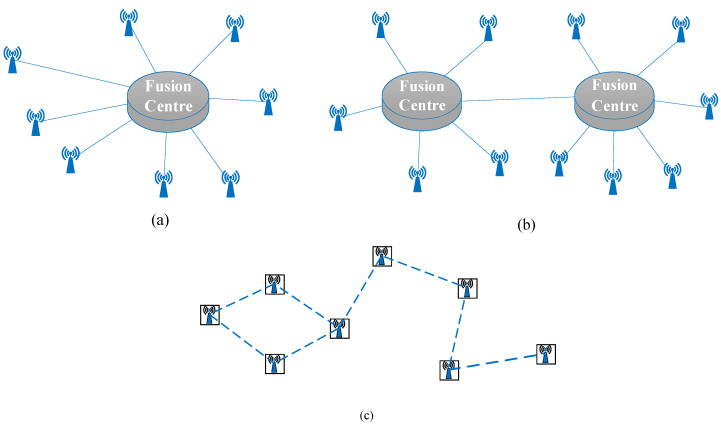
(**a**) Centralized fusion, (**b**) hierarchical fusion, and (**c**) decentralized fusion architectures.

Because the detection accuracy of a multi-sensor network is higher than that of a single sensor, the appropriate sensor network structure is generally selected for data fusion. In general, the multi-sensor data fusion framework is divided into centralized, decentralized, and hierarchical architecture [31], as shown in Figure 4. In the centralized fusion framework (Figure 4a), all sensors communicate with a fusion center through single-hop or multi-hop communication. Although there is no loss in measurement, it increases the computational burden of the fusion center. Distinct from the centralized framework, the hierarchical fusion framework has multiple fusion centers, sensors are grouped in clusters, each having a fusion center, and fusion centers communicate each other, which improves the data backup ability but also increases the communication burden of low-cost sensors (Figure 4b). When all sensors communicate with each other without a fusion center (Figure 4c), this is termed a decentralized fusion architecture.

**Figure 5:**
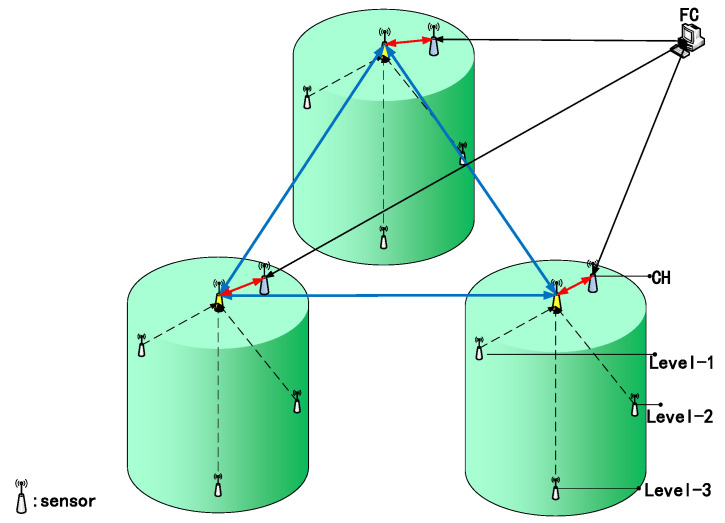
Hierarchical data fusion framework for clustered multi-connected sensor networks.

In the multistage oxidation tank scene in the biological oxidation pretreatment process, the traditional temperature sensor measurement position is generally located at the top of the device, so it is difficult to detect the temperature change at the edge of the oxidation tank. This method has serious limitations. Considering the uneven temperature distribution in extreme environments, we designed a hierarchical data fusion framework for clustered multi-connection sensor networks used in multi-stage oxidation tanks. As shown in Figure 5, the fusion framework is divided into three layers and consists of **N** sensors.

According to the distribution of the first three-stage oxidation tank, it is divided into three clusters, and each cluster is divided into two stages for local fusion estimation. Generally, the fusion framework was designed based on the principle of accurate measurement of the local area and complete coverage of the whole area. The pulp temperature of all oxidation tanks can be measured effectively, and the global temperature change can be effectively obtained after data processing by the fusion algorithm description.

## 3. Algorithm Description

### 3.1. Local State Estimation

Consider the hierarchical fusion framework of clustered multi-connected sensor networks as shown in Figure 5, where the state of the pulp temperature to be estimated is described by the following discrete-time state-space model:

(6) $$X_{k+1}=A_{k}X_{k}+\omega_{k},k=0,1,......$$

The measurement equation of the sensor inside the oxidation tank at all levels is given by

(7) $$Z_{\left(i,k\right)}=C_{\left(i,k\right)}X_{k}+v_{\left(i,k\right)},i=1,2,\ldots ,N$$

where $X_{k}$ represents the pulp temperature state vector in the oxidation tank and $\omega_{k}$ is the system state noise, which is usually assumed to be zero-mean Gaussian white noise with a non-negative definite covariance matrix $Q_{k}$, and $v_{\left(i,k\right)}$ represents the observation noise of the *i*th sensor, which is usually assumed to be zero-mean Gaussian white noise with positive definite covariance matrix $R_{k}$.

**Figure 6:**
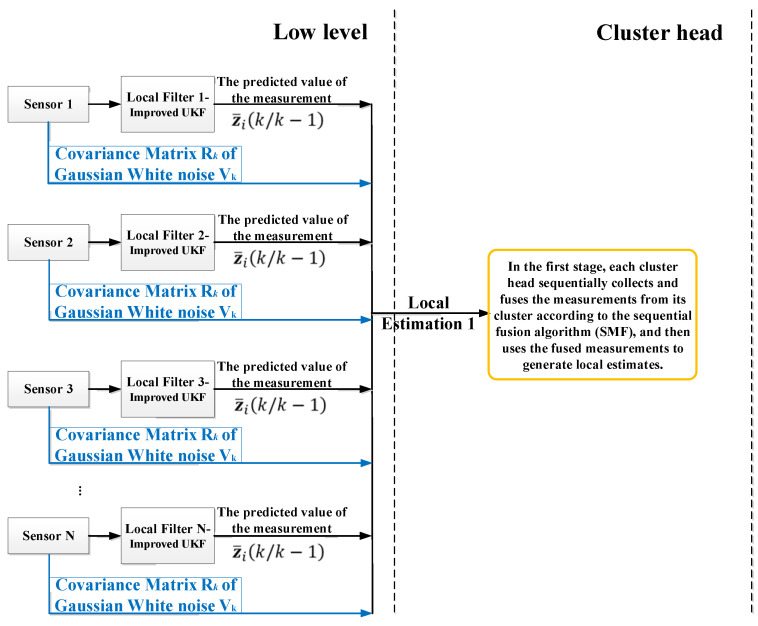
Framework of local fusion estimation algorithm.

The two-stage framework of the proposed local fusion estimation algorithm is shown in Figure 6. When the bottom sensor in the cluster (that is, inside each oxidation tank) collects the original pulp temperature data, the improved UKF algorithm is introduced to improve the performance of local state estimation. At the cluster head, according to the characteristics of the sequential measurement fusion algorithm (SMF) and the situation of each local filter at the bottom, the fusion link with the measurement update after the UT transform is established, and the measurement estimation of local fusion is obtained.

#### 3.1.1. Traditional UKF Algorithm

Before describing the improved UKF, let us briefly familiarize ourselves with the traditional UKF for the previously analyzed nonlinear system model (6). The overview of the traditional UKF implementation process is as follows:

Step 1: Initialization. Assume that the state estimation and error covariance of the system are respectively ${\bar{x}}_{i}\left(k\right)$ and $P_{i}\left(k\right)$.

(8) $$\begin{array}{l} {\bar{x}}_{i}\left(0\right)=E\left\{x_{i}\left(0\right)\right\}\\ P_{i}\left(0\right)=E\left\{\left[x_{i}\left(0\right)-{\bar{x}}_{i}\left(0\right)\right]{\left[x_{i}\left(0\right)-{\bar{x}}_{i}\left(0\right)\right]}^{T}\right\} \end{array}$$

Step 2: Acquire the sampling points and calculate the corresponding weight to approximate the *n*-dimensional state vector x with mean and covariance.

(9) $$x_{i\left(k-1\right)}^{\left(0\right)}={\bar{x}}_{i}\left(k-1\right)$$

(10) $$x_{i\left(k-1\right)}^{\left(j\right)}={\bar{x}}_{i}\left(k-1\right)+{\left(\sqrt{\left(n+\sigma \right)P_{i}\left(k-1\right)}\right)}_{j},j=1,\ldots ,n$$

(11) $$x_{i\left(k-1\right)}^{\left(j\right)}={\bar{x}}_{i}\left(k-1\right)-{\left(\sqrt{\left(n+\sigma \right)P_{i}\left(k-1\right)}\right)}_{j},j=n+1,\ldots ,2n$$

(12) $$W_{0}^{m}=W_{j}^{c}=W_{j+n}=\{\begin{array}{l} \sigma /n+\sigma ,\;\;\;\;\;\;\;\;\;j=0\\ 1/2\left(n+\sigma \right),\;\;\;j\neq 0 \end{array}$$

Here, ${\left(\sqrt{\left(n+\sigma \right)P_{i}\left(k-1\right)}\right)}_{j}$ represents the square root of the matrix $\left(n+\sigma \right)P_{i}\left(k-1\right)$ column *j* or row *j*, the superscript m is the mean of the scalar weight *W*, c is the covariance, and the subscript is the sampling point sequence number. The parameter σ is a scaling parameter, which is used to reduce the total prediction error, and n+σ=3 is available for Gaussian distribution.

Step 3: Prediction procedure:

(13) $$x_{i\left(k/k-1\right)}^{\left(j\right)}=f\left(x_{i\left(k-1\right)}^{\left(j\right)}\right)\;\;\;\;\;\;\left(j=0,1,\ldots ,2n\right),$$

(14) $${\bar{x}}_{i}\left(k/k-1\right)=\sum_{j=0}^{2n}W_{j}^{m}\left(x_{i\left(k/k-1\right)}^{\left(j\right)}\right),$$

(15) $$P_{i}\left(k/k-1\right)=\sum_{j=0}^{2n}W_{j}^{c}\left(x_{i\left(k/k-1\right)}^{\left(j\right)}-{\bar{x}}_{i}\left(k/k-1\right)\right){\left(x_{i\left(k/k-1\right)}^{\left(j\right)}-{\bar{x}}_{i}\left(k/k-1\right)\right)}^{T}+Q_{k}.$$

Step 4: According to the one-step predicted value, the UT transform is carried out again to produce a new set of sigma points:

(16) $${x^{\prime }}_{i\left(k/k-1\right)}^{\left(0\right)}={\bar{x}}_{i}\left(k/k-1\right),$$

(17) $${x^{\prime }}_{i\left(k/k-1\right)}^{\left(j\right)}={\bar{x}}_{i}\left(k/k-1\right)+{\left(\sqrt{\left(n+\sigma \right)P_{i}\left(k/k-1\right)}\right)}_{j^{\prime }},j^{\prime }=1,\ldots ,n,$$

(18) $${x^{\prime }}_{i\left(k/k-1\right)}^{\left(j\right)}={\bar{x}}_{i}\left(k/k-1\right)-{\left(\sqrt{\left(n+\sigma \right)P_{i}\left(k/k-1\right)}\right)}_{j^{\prime }},j^{\prime }=n+1,\ldots ,2n.$$

Step 5: Measurement update:

(19) $$z_{i\left(k/k-1\right)}=C\left({x^{\prime }}_{i\left(k/k-1\right)}^{\left(j\right)}\right),$$

(20) $${\bar{z}}_{i}\left(k/k-1\right)=\sum_{j=0}^{2n}W_{j}^{m}\left[z_{i\left(k/k-1\right)}\right],$$

(21) $$P_{i}\left({\bar{z}}_{i}\left(k/k-1\right)\right)=\sum_{j=0}^{2n}W_{j}^{c}\left(z_{i\left(k/k-1\right)}-{\bar{z}}_{i}\left(k/k-1\right)\right){\left(z_{i\left(k/k-1\right)}-{\bar{z}}_{i}\left(k/k-1\right)\right)}^{T}+R_{k},$$

(22) $$P_{i}\left({\bar{x}}_{i}\left(k/k-1\right){\bar{z}}_{i}\left(k/k-1\right)\right)=\sum_{j=0}^{2n}W_{j}^{c}\left(x_{i\left(k/k-1\right)}^{\left(j\right)}-{\bar{x}}_{i}\left(k/k-1\right)\right){\left(z_{i\left(k/k-1\right)}-{\bar{z}}_{i}\left(k/k-1\right)\right)}^{T}.$$

The filter gain is given by

(23) $$K_{i}\left(k\right)=P_{i}\left({\bar{x}}_{i}\left(k/k-1\right){\bar{z}}_{i}\left(k/k-1\right)\right)P_{i}^{-1}\left({\bar{z}}_{i}\left(k/k-1\right)\right).$$

Calculate the status update and covariance update of the system:

(24) $${\bar{x}}_{i}\left(k\right)={\bar{x}}_{i}\left(k/k-1\right)+K_{i}\left(k\right)\left(z_{i\left(k\right)}-{\bar{z}}_{i}\left(k/k-1\right)\right),$$

(25) $$P_{i}\left(k\right)=P_{i}\left(k/k-1\right)-K_{i}\left(k\right)P_{i}\left({\bar{z}}_{i}\left(k/k-1\right)\right)K_{i}^{T}\left(k\right).$$

Step 6: Repeat Steps 2 to 5 to carry out the next time filtering calculation.

#### 3.1.2. The Improved UKF

The UKF algorithm abandons the traditional step of linearizing the nonlinear function, uses the unscented transform (UT) to determine the sampling point near the estimation point, and calculates the mean and covariance for the one-step prediction equation. Thus, the estimation accuracy of UKF depends on the accuracy of the mean and covariance calculated by UT. UKF does not need to calculate the Jacobian matrix like the extended Kalman filter (EKF), and UKF is better than EKF for the same amount of computation. However, the UKF also has some shortcomings: (1) There is a problem with identifying process modeling errors in nonlinear multi-sensor systems; (2) because the fixed values of covariance matrices $Q_{k}$ and $R_{k}$ cannot reflect the actual characteristics of system errors and measurement errors, there is a problem with random errors. 

**Figure 7:**
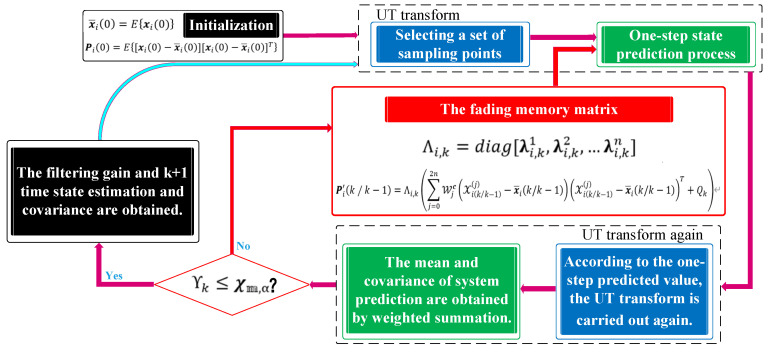
Procedure of improved unscented Kalman filtering.

If the assumptions related to (6) and (7) are true, there is the following probability density function [32]:

(26) p(ℤ˜i(k))=N(ℤ˜i(k);0,Pi(z¯i(k/k−1)))=(−12(ℤ˜i(k))T(Pi(z¯i(k/k−1)))−1(ℤ˜i(k)))((2π)m|Pi(z¯i(k/k−1))|)

where ${\tilde{\mathbb{Z}}}_{i\left(k\right)}=z_{i\left(k\right)}-{\bar{z}}_{i}\left(k/k-1\right)$ denotes the innovation vector of the *i*th local filter and $\left|P_{i}\left({\bar{z}}_{i}\left(k/k-1\right)\right)\right|$ is the determinant of $P_{i}\left({\bar{z}}_{i}\left(k/k-1\right)\right)$. If the above formula is false, it is determined that there is a nonlinear system modeling error, and the criterion for detecting whether there is a modeling error or not can be further used, in which the square root of the negative index represents the Mahalanobis distance between ${\tilde{\mathbb{Z}}}_{i\left(k\right)}$ and the zero vector.

(27) $${ϒ}_{k}=M_{i\left(k\right)}^{2}={\left(\sqrt{{\left({\tilde{\mathbb{Z}}}_{i\left(k\right)}\right)}^{T}{\left(P_{i}\left({\bar{z}}_{i}\left(k/k-1\right)\right)\right)}^{-1}\left({\tilde{\mathbb{Z}}}_{i\left(k\right)}\right)}\right)}^{2}={\left({\tilde{\mathbb{Z}}}_{i\left(k\right)}\right)}^{T}{\left(P_{i}\left({\bar{z}}_{i}\left(k/k-1\right)\right)\right)}^{-1}\left({\tilde{\mathbb{Z}}}_{i\left(k\right)}\right),$$

where ${ϒ}_{k}$ obeys the Chi-square distribution with degree of freedom 𝕞 for a given significance level α, (0<α<1).

(28) $$\mathrm{Pr}=\{\begin{array}{c} \alpha \;\;\;\;\;\;\;\;{ϒ}_{k}>\chi_{𝕞,\alpha }\\ 1-\alpha \;\;\;\;\;\;{ϒ}_{k}\leq \chi_{𝕞,\alpha }\;\; \end{array}$$

where the α-quantile $\chi_{𝕞,\alpha }$ of the chi-square distribution is predetermined. The above formula represents random probability events, and if ${ϒ}_{k}$$\leq \chi_{𝕞,\alpha }$ (Pr=1−α) is established, there is no nonlinear system modeling error; conversely, there is a process modeling error in high probability in multi-sensor systems. Given this situation, the traditional UKF needs to be improved and the fading memory matrix $\Lambda_{i,k}$ is introduced.

(29) $${P^{\prime }}_{i}\left(k/k-1\right)=\Lambda_{i,k}\left(\sum_{j=0}^{2n}W_{j}^{c}\left(x_{i\left(k/k-1\right)}^{\left(j\right)}-{\bar{x}}_{i}\left(k/k-1\right)\right){\left(x_{i\left(k/k-1\right)}^{\left(j\right)}-{\bar{x}}_{i}\left(k/k-1\right)\right)}^{T}+Q_{k}\right)$$

where $\Lambda_{i,k}=diag\left[\lambda_{i,k}^{1},\lambda_{i,k}^{2},\ldots ,\lambda_{i,k}^{n}\right],\;\lambda_{i,k}^{j}\geq 1,j=1,2,\ldots ,n$ are the fading factors. The fading memory matrix $\Lambda_{i,k}$ corrects the covariance matrix of the predicted state and finally sets-up ${ϒ}_{k}$$\leq \chi_{𝕞,\alpha }$. The steps of the improved UKF algorithm are shown in Figure 7.

#### 3.1.3. Embedding of Sequential Measurement Fusion

In this section, we design a reasonable local fusion estimator for the second stage sensor in each cluster (inside the oxidation tank). After filtering the original temperature data using the underlying sensor in the first stage, the idea of sequential measurement fusion is embedded into the measurement update link after the UT in the improved UKF, and the measurements sent in time series are fused. Local measurement estimates are then generated. 

**Figure 8:**
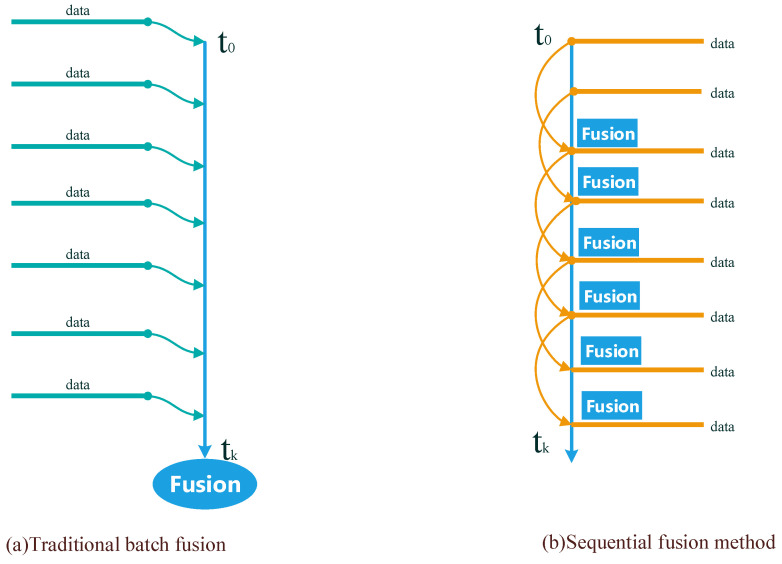
Comparison between (**a**) traditional batch fusion and (**b**) sequential fusion methods.

Sequential analysis was first proposed by Abraham Wolde in 1940 as a method to test statistical hypotheses and continuously analyze statistical data on the premise that they are available [33]. After that, it was widely used in the field of signal detection. As asynchronous transmission often occurs in sensor networks, the traditional batch processing fusion algorithm does not provide advantages in computational complexity and running time, whereas the sequential fusion method carries out real-time fusion according to the time order in which each sensor datum arrives at the estimator. As shown in Figure 8, the accuracy of the batch processing fusion algorithm is the same as that of the sequential fusion algorithm. Especially for low power consumption and time-determined applications, it is more desirable than the traditional fusion algorithm.

Consider that the sensor measurements arrive at the processing center in arbitrary order, and without losing generality, assume that the order is indicated by the sensor number. For the measurement update value M filtered by the improved UKF algorithm, the sequential observation fusion estimator (SOFE) is given by the following equation:

(30) $$R_{i\left(\lambda \right)}^{SOF}=\{\begin{array}{l} \left(R_{i\left(\lambda -1\right)}^{-1}+R_{i\left(\lambda +1\right)}^{-1}\right)\;\;\;\;\;\;\;\;\;\;\;\;,\;\;\;\;\;\;\;\;\;{\bar{z}}_{i}\left(k/k-1\right)\geq \psi_{th}\\ \left(R_{i\left(\lambda -1\right)}^{-1}\xi_{i,k}+R_{i\left(\lambda +1\right)}^{-1}\xi_{i,k}\right)\;\;,\;\;\;\;\;\;\;\;\;{\bar{z}}_{i}\left(k/k-1\right)<\psi_{th} \end{array}$$

(31) $$Z_{i\left(\lambda \right)}^{SOF}={\left(R_{i\left(\lambda \right)}^{SOF}\right)}^{-1}\{\begin{array}{l} \left(R_{i\left(\lambda -1\right)}^{-1}{\bar{z}}_{i}\left(\lambda -1\right)+R_{i\left(\lambda +1\right)}^{-1}{\bar{z}}_{i}\left(\lambda -1\right)\right)\;\;\;\;\;\;\;\;\;\;\;\;,\;\;\;\;\;\;\;\;\;{\bar{z}}_{i}\left(k/k-1\right)\geq \psi_{th}\\ \left(R_{i\left(\lambda -1\right)}^{-1}\xi_{i,k}{\bar{z}}_{i}\left(\lambda -1\right)+R_{i\left(\lambda +1\right)}^{-1}\xi_{i,k}{\bar{z}}_{i}\left(\lambda +1\right)\right)\;\;,\;\;\;\;\;\;\;\;\;{\bar{z}}_{i}\left(k/k-1\right)<\psi_{th} \end{array}$$

where $Z_{i\left(\lambda \right)}^{SOF}$ and $R_{i\left(\lambda \right)}^{SOF}$ represent the result of the fusion of observation and observation noise covariance, respectively; λ=1,…,n−1 represents the sequence of sensor sampling data in each cluster sent to the local fusion estimator; and $\xi_{i,k}$ is the empirical correction matrix of the sensor measurement in the oxidation tank, which is affected by the change in spatial position and temperature field. Considering the sudden temperature drop of the upwind position in the oxidation tank in an extreme environment, $\psi_{th}$ is set as the temperature measurement threshold. The divergence of the local fusion process is suppressed by correcting the noise covariance matrix $R_{k}$.

### 3.2. Global State Fusion Estimation

Without losing generality, we established the global fusion center of all clusters (that is, three oxidation tanks) to achieve global optimal fusion estimation. A sequential analysis and inverse covariance intersection-global state fusion estimation (SICI-GSFE) based on sequential analysis and inverse covariance intersection is proposed. In the third stage, after collecting the state estimation data from all clusters, the proposed SICI-GSFE algorithm is used for global state fusion estimation to reduce the noise between local estimators and improve the performance of global fusion estimation. The idea of covariance intersection originates from the use of the convex combination of the mean and covariance of variables to propose a covariance intersection algorithm for random variables with an unknown degree of cross-correlation [34]. After that, ideas of ellipsoid covariance intersection and inverse covariance intersection were derived, which are more suitable to be combined with sequential analysis and applied to the state fusion estimation algorithm.

${\bar{x}}_{i}\left(k\right)$ is the unbiased estimation of the state equation of pulp temperature in the oxidation tank and $P_{i}\left(k\right)$ represents the estimation error covariance matrix of ${\bar{x}}_{i}\left(k\right)$. Then, SICI-GSFE is given by the following equation for any Θ∈[0,1]:

(32) $$\Gamma_{SICI}=P_{SICI}\cdot \left(P_{i\left(\lambda -1\right)}^{-1}-\Theta {\left(\Theta P_{i\left(\lambda -1\right)}+\left(1-\Theta \right)P_{i\left(\lambda +1\right)}\right)}^{-1}\right),$$

(33) $$\Psi_{SICI}=P_{SICI}\cdot \left(P_{i\left(\lambda +1\right)}^{-1}-\left(1-\Theta \right){\left(\Theta P_{i\left(\lambda -1\right)}+\left(1-\Theta \right)P_{i\left(\lambda +1\right)}\right)}^{-1}\right).$$

After the corresponding weights are calculated, the final global fusion result is obtained:

(34) $${\bar{X}}_{i\left(\lambda \right)}^{SICIF}=\Gamma_{SICI}{\bar{x}}_{i\left(\lambda -1\right)}+\Psi_{SICI}{\bar{x}}_{i\left(\lambda +1\right)},$$

(35) $$P_{SICI}^{-1}=P_{i\left(\lambda -1\right)}^{-1}+P_{i\left(\lambda +1\right)}^{-1}-{\left(\Theta P_{i\left(\lambda -1\right)}+\left(1-\Theta \right)P_{i\left(\lambda +1\right)}\right)}^{-1},$$

where is the adjustable parameter; Θ represents the weight assigned to the covariance of the estimation error, which can be optimized according to different performance indicators; and $\Gamma_{SICI}$, $\Psi_{SICI}$ represent the fusion weight, which satisfies the following relationship:

(36) $$\Gamma_{SICI}+\Psi_{SICI}=I,$$

(37) $$Cov\left[\left(\Gamma_{SICI}{\bar{x}}_{i\left(\lambda -1\right)}+\Psi_{SICI}{\bar{x}}_{i\left(\lambda +1\right)}-X_{k}\right){\left(\Gamma_{SICI}{\bar{x}}_{i\left(\lambda -1\right)}+\Psi_{SICI}{\bar{x}}_{i\left(\lambda +1\right)}-X_{k}\right)}^{T}\right],$$

(38) $$=Cov\left[\left(\Gamma_{SICI}{\tilde{X}}_{i\left(\lambda -1\right)}+\Psi_{SICI}{\tilde{X}}_{i\left(\lambda +1\right)}\right){\left(\Gamma_{SICI}{\tilde{X}}_{i\left(\lambda -1\right)}+\Psi_{SICI}{\tilde{X}}_{i\left(\lambda +1\right)}\right)}^{T}\right],$$

(39) $$\begin{array}{l} =\Gamma_{SICI}P_{i\left(\lambda -1\right)}\Gamma_{SICI}^{T}+\Psi_{SICI}P_{i\left(\lambda +1\right)}\Psi_{SICI}^{T}+\Gamma_{SICI}P_{i\left(\lambda -1\right)}\Omega^{-1}P_{i\left(\lambda +1\right)}\Psi_{SICI}^{T}\\ +\Psi_{SICI}P_{i\left(\lambda +1\right)}\Omega^{-1}P_{i\left(\lambda -1\right)}\Gamma_{SICI}^{T} \end{array}$$

where I represents the unit matrix corresponding to the appropriate dimension, ${\tilde{X}}_{i\left(\lambda -1\right)}$ and ${\tilde{X}}_{i\left(\lambda +1\right)}$ are the state estimation errors of each sensor based on the time series, and Ω indicates that the degree of redundancy estimation between sensors is parameterized.

## 4. Simulation and Discussion

To prove the effectiveness of the proposed local sequential measurement fusion estimation and global state fusion estimation algorithm, taking the multi-stage oxidation tank pretreatment process of a biological oxidation gold extraction plant in Xinjiang as the example, the oxidation tank reaction environment was simulated in the laboratory, and MATLAB software version R2016a (MathWorks in Nadik, MA, USA) was used to simulate the fusion algorithm. The bacteria used in the biological oxidation pretreatment technology are mainly autotrophic bacteria that need oxidation energy [35]. To ensure its best activity, the optimum temperature in actual production was set to 42 °C.

**Figure 9:**
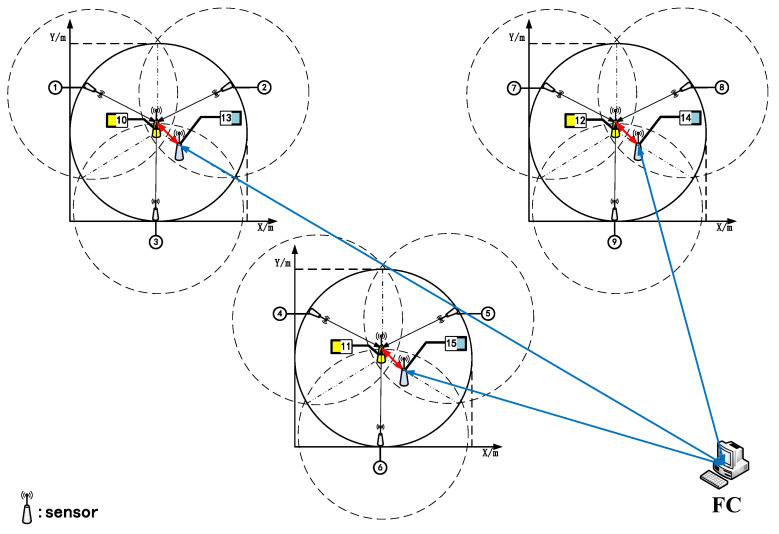
Clustering distributed sensor network (FC: Fusion center).

As shown in Figure 9, sensors 1–9 in the first stage represent the first-level sensors for measuring the temperature in the area where the oxidation tank is vulnerable to environmental interference. The temperature data measured by this sensor provide the basis for the fusion algorithm and reflect the changing trend in pulp temperature in the oxidation tank. In the second stage, sensors 10–12 collect and fuse the measured values in a sequential manner to generate local fusion estimates. Sensors 13–15 send the estimated data of each cluster to the global fusion center (the third level) on the basis of communicating with each other to generate global fusion state estimates.

In the process of numerical simulation, the structure of the sensor network follows the framework shown in Figure 5. Figure 2 shows the uneven distribution of the temperature field in the biological oxidation tank under different climatic conditions and the difference in the position and the working level of the sensors in the network, so the parameters related to the observation noise differ. To evaluate the simulation results strictly, three performance indexes, root-mean-square error (RMSE), mean absolute error (MAE), and mean relative error (MRE), were used to evaluate the algorithm. The calculation equations are as follows:

(40) $$RMSE=\sqrt{\frac{1}{N}\sum_{k=1}^{N}{\left({\bar{X}}_{i\left(k,\lambda \right)}^{F}-X_{i\left(k\right)}\right)}^{2}},$$

(41) $$MAE=\frac{1}{N}\sum_{k=1}^{N}\left|{\bar{X}}_{i\left(k,\lambda \right)}^{F}-X_{i\left(k\right)}\right|,$$

(42) $$MRE=\frac{1}{N}\sum_{k=1}^{N}\frac{\left|{\bar{X}}_{i\left(k,\lambda \right)}^{F}-X_{i\left(k\right)}\right|}{X_{i\left(k\right)}}.$$

**Figure 10:**
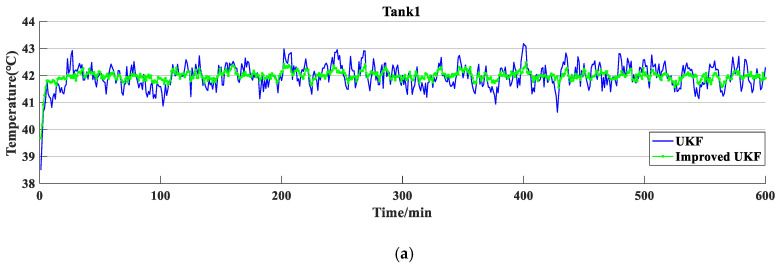

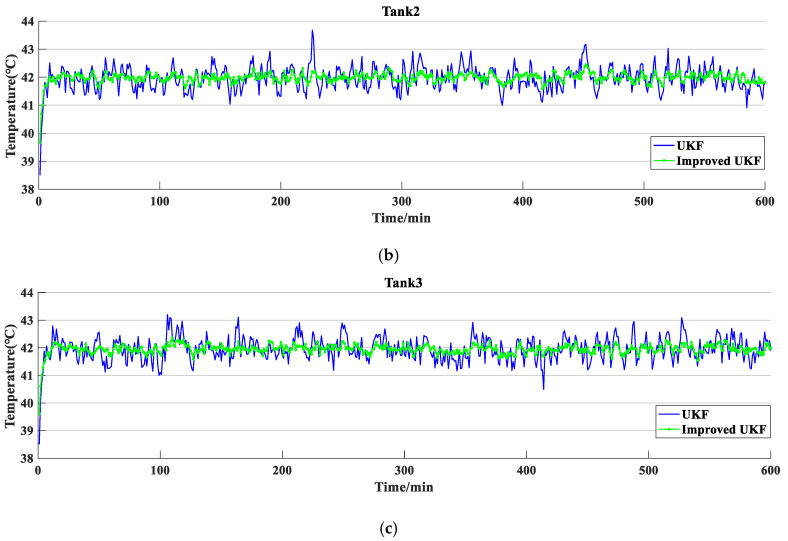
Comparison of simulation results between traditional and improved unscented Kalman filter (UKF) in (**a**) Tank1, (**b**) Tank2, and (**c**) Tank3.

**Figure 11:**
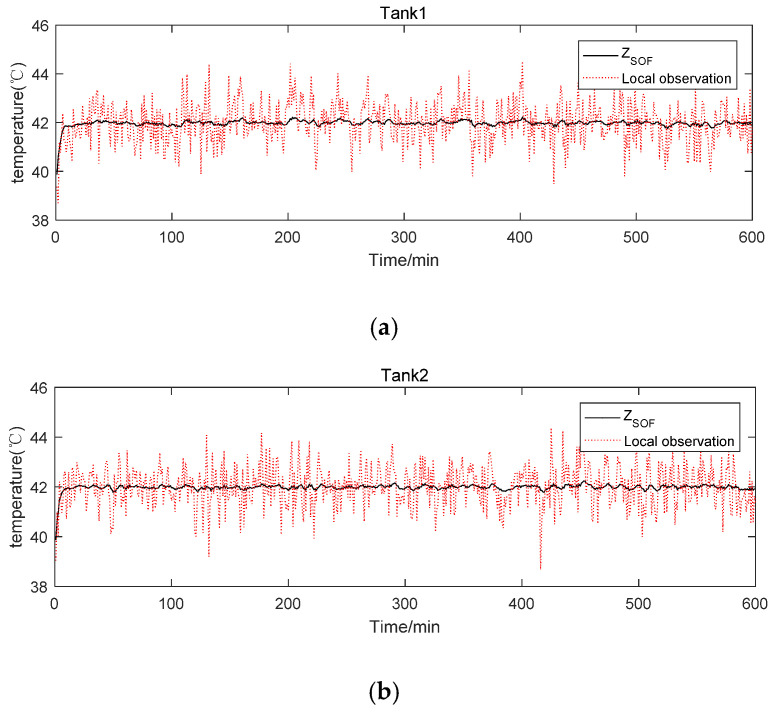

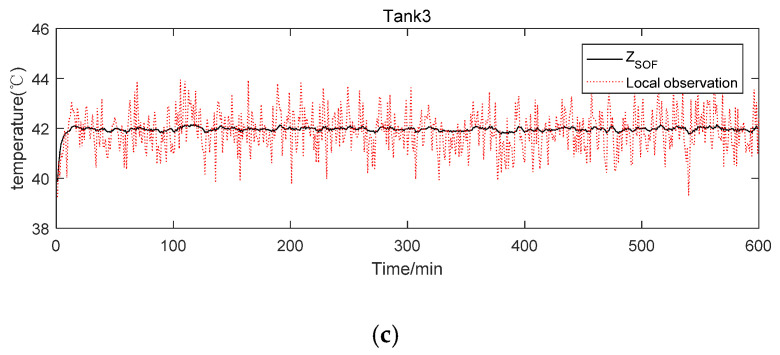
Simulation results of local measurement fusion estimation in each cluster: (**a**) Tank1, (**b**) Tank2, (**c**) Tank3.

First, the results of local fusion estimation in each cluster were simulated, as shown in Figure 10, and the results showed that the volatility of the improved UKF is lower than that of the traditional UKF. Figure 11 shows the temperature curve of the oxidation tank simulated by the local fusion SOFE algorithm in each cluster. Compared to the observed curve of the underlying sensor, the fused temperature curve is more stable. Table 2 shows that the running time of the SOFE algorithm is only 1.972425 s, which is more efficient than the 4.765901 s of the general weighted batch fusion (BF) algorithm.

**Figure 12:**
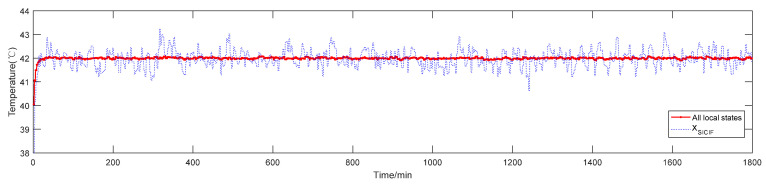
Simulation results of sequential analysis and inverse covariance intersection-global state fusion estimation (SICI-GSFE) algorithm.

**Figure 13:**
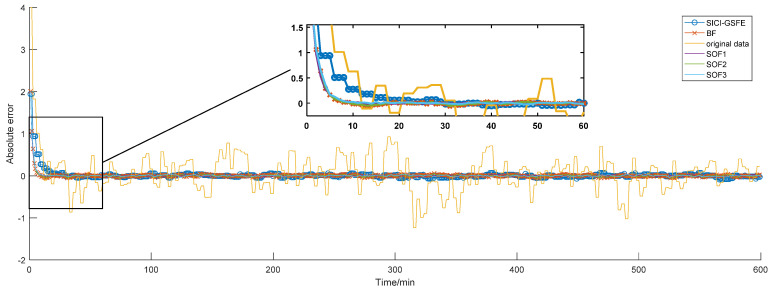
Absolute error of local measurement fusion, general batch fusion, and global fusion state estimation.

From the perspective of global state fusion estimation, Figure 12 shows the global state estimation curves of the three clusters obtained by the SICI-GSFE algorithm. Compared to the underlying state estimation, the volatility is significantly lower. Figure 13 shows the absolute error of the simulation results of the SOFE algorithm, the BF algorithm, and the SICI-GSFE algorithm. Table 3 lists the metrics of the three evaluation performance metrics for each algorithm.

## 5. Conclusions

The goal of this research was to improve the accuracy of pulp temperature state estimation of the primary oxidation tank in the biological oxidation gold extraction process to propose a clustered hierarchical sensor network fusion algorithm that can adapt to the variations in high-altitude environments. The detailed principle of heat transfer in the oxidation tank was introduced, and a reasonable framework of local measurement fusion and global state fusion of multi-connected sensors was designed. In the data processing of the local bottom sensor, the decay memory matrix was added on the basis of the traditional UKF algorithm, and the sequential fusion algorithm was embedded in the measurement update to improve the identification of nonlinear modeling errors. The fusion accuracy of the sequential fusion algorithm was also improved, and the two complement each other. The local measurement sequential observation fusion (SOF) with a filtering effect was found to be more computationally efficient than the general batch fusion algorithm. The global state fusion estimation algorithm SICI-GSFE increases the accuracy of global fusion state estimation by combining the idea of the inverse covariance intersection with sequential analysis. The effectiveness of the proposed algorithm was verified by computer simulation. Future research work will be divided into two aspects: Exploring the applicability of sensor networks designed in different scenarios that meet the conditions of clustering and hierarchical space, and the impact of the proposed fusion algorithm on different detection targets, such as large grain reserves, and temperature and humidity detection in warehouses; second, after improving the accuracy of target monitoring, researching a local control strategy based on global sensor network monitoring, and establishing a distributed fusion center decision feedback control system.

## Figures and Tables

**Table 1:** Notation used in this study.

Notation	Description	Notation	Description
β	heat conduction coefficient	$P_{i}\left(k\right)$	error covariance of the system
$\Psi_{h}$	temperature of the serpentine heat exchanger	σ	a scaling parameter
$\Psi_{o}$	temperature of the pulp	$K_{i}\left(k\right)$	filter gain
h	heat transfer coefficient	$x_{i\left(k-1\right)}^{\left(j\right)}$	sampling points
ρ	density	$x_{i\left(k/k-1\right)}^{\left(j\right)}$	one-step predicted value
ℂ	specific heat capacity	${x^{\prime }}_{i\left(k/k-1\right)}^{\left(j\right)}$	a new set of sigma points
γ	heat source (heat exchanger)	${\bar{z}}_{i}\left(k/k-1\right)$	measurement update value
ω	temperature white noise	$P_{i}\left({\bar{z}}_{i}\left(k/k-1\right)\right)$	error covariance of the measurement
$Q_{l}$	heat loss per unit area per unit time	${\tilde{\mathbb{Z}}}_{i\left(k\right)}$	the innovation vector of the ith local filter
$\eta_{k}$	white noise at the heat source	${ϒ}_{k}$	obeys chi-square distribution with degree of freedom 𝕞
$v_{\left(i,k\right)}$	observation noise of the ith sensor	α	a given significance level
$Q_{k}$	system state noise covariance matrix	$\chi_{𝕞,\alpha }$	chi-square distribution
$R_{k}$	observation noise covariance matrix	$\Lambda_{i,k}$	fading memory matrix
${\bar{x}}_{i}\left(k\right)$	unbiased estimation of the state	λ	sequence of sensor sampling data in each cluster
$\xi_{i,k}$	empirical correction matrix	$\psi_{th}$	temperature measurement threshold
${\bar{X}}_{i\left(\lambda \right)}^{SICIF}$	final global fusion result	Θ	weight assigned to the covariance of the estimation error
$\Gamma_{SICI}$, $\Psi_{SICI}$	fusion weight	${\tilde{X}}_{i\left(\lambda -1\right)}$	state estimation errors of each sensor based on time series
I	unit matrix corresponding to the appropriate dimension	Ω	degree of redundancy estimation between sensors is parameterized

**Table 2:** The running time of the sequential observation fusion estimator (SOFE) algorithm and the general weighted batch fusion (BF) algorithm.

Type	Running Time (s)
SOFE	1.972425
BF	4.765901

**Table 3:** Comparison of performance indicators of local measurement fusion estimation, general batch fusion method in each cluster, and global state fusion estimation.

Type	Performance Index		
	(Root-Mean-Square Error) RMSE (°C)	(Mean Absolute Error) MAE (°C)	(Mean Relative Error) MRE (%)
SOFE (1#tank)	0.0953	0.0168	0.04
SOFE (2#tank)	0.0994	0.0222	0.05
SOFE (3#tank)	0.0990	0.0194	0.05
BF	0.0991	0.0224	0.05
SICI-GSFE	0.1408	0.0399	0.09
